# Species Distribution 2.0: An Accurate Time- and Cost-Effective Method of Prospection Using Street View Imagery

**DOI:** 10.1371/journal.pone.0146899

**Published:** 2016-01-11

**Authors:** Laurent Hardion, Agathe Leriche, Eugénie Schwoertzig, Alexandre Millon

**Affiliations:** 1 Institut Méditerranéen de Biodiversité et d’Ecologie marine et continentale (IMBE), Aix Marseille Université, CNRS, IRD, Avignon Université, 13331 Marseille, France; 2 Laboratoire Image Ville Environnement (LIVE), Université de Strasbourg, CNRS, 67000 Strasbourg, France; National University of Mongolia, MONGOLIA

## Abstract

Species occurrence data provide crucial information for biodiversity studies in the current context of global environmental changes. Such studies often rely on a limited number of occurrence data collected in the field and on pseudo-absences arbitrarily chosen within the study area, which reduces the value of these studies. To overcome this issue, we propose an alternative method of prospection using geo-located street view imagery (SVI). Following a standardised protocol of virtual prospection using both vertical (aerial photographs) and horizontal (SVI) perceptions, we have surveyed 1097 randomly selected cells across Spain (0.1x0.1 degree, *i*.*e*. 20% of Spain) for the presence of *Arundo donax* L. (Poaceae). In total we have detected *A*. *donax* in 345 cells, thus substantially expanding beyond the now two-centuries-old field-derived record, which described *A*. *donax* only 216 cells. Among the field occurrence cells, 81.1% were confirmed by SVI prospection to be consistent with species presence. In addition, we recorded, by SVI prospection, 752 absences, *i*.*e*. cells where *A*. *donax* was considered absent. We have also compared the outcomes of climatic niche modeling based on SVI data against those based on field data. Using generalized linear models fitted with bioclimatic predictors, we have found SVI data to provide far more compelling results in terms of niche modeling than does field data as classically used in SDM. This original, cost- and time-effective method provides the means to accurately locate highly visible taxa, reinforce absence data, and predict species distribution without long and expensive *in situ* prospection. At this time, the majority of available SVI data is restricted to human-disturbed environments that have road networks. However, SVI is becoming increasingly available in natural areas, which means the technique has considerable potential to become an important factor in future biodiversity studies.

## Introduction

Species occurrence data represent basic but indispensable information for research in conservation biology, biogeography, ecology, and evolution [1]. These data provide guidance for sampling design, but they also provide a basis for planning geographical areas for conservation policies [2], for studying spatial dynamics and risks of invasive species [3], or for defining biogeographical patterns according to environmental variables [4]. Over the last 20 years, occurrence data have been increasingly valued with the development of species distribution models (SDM) as predictive tools in the context of global change [5, 6]. Based on accurate species occurrences, SDM can indeed predict the presence probabilities of individuals in uninformed areas on the basis of relevant environmental predictors [7]. In the framework of biological invasions, this tool makes it possible to not only model the ecological niche of pests or weeds in their native range and extrapolate to potential areas of risk [8, 9], but also to identify the geographical origin of an overlooked invasive species based on its introduced range [10].

Species occurrence data are available in various forms. For plant species, herbarium collections and botanical literature have remained the main sources of plant distribution data for centuries. More recently, the development of online databases, which gather contributions not only from scientists but also from informed citizens [11], has allowed several countries to provide accurate gauges of species occurrence within their borders. On a broader scale, the Global Biodiversity Information Facility project (GBIF, www.gbif.org) collects freely available distribution data worldwide. However, the accuracy of these occurrence data is heterogeneous. To begin with, the data generally suffer of a lack of completeness, particularly when it is sourced from developing countries [12]. In addition, most online databases are not subject to thorough verification. As an example, Yesson *et al*. rejected 16% of the occurrence data for legume species included in the GBIF database [13]. The erroneous data comprised such a high proportion of the total that, had it not been rejected, it would have been all but impossible to obtain a reliable niche estimate via SDM modelling. Besides, unresolved taxonomy and synonymous nomenclature add another layer of bias to species distribution data [14]. For example, the analyse of 4500 specimens of ginger species across 40 international herbaria revealed that at least 58% of them were misidentified or not updated regarding recent taxonomy [15]. Overall, the main limitation of such large-scale data lies in the lack of information about the spatial sampling design and effort, and the unavailability of supported absence data.

Indirect methods using photo-interpretation or remote sensing on aerial photographs have been developed to collect continuous data on vegetation cover. These methods use spectral signals to define rough contours of vegetation patches and habitats, but they rarely allow aerial identification of plant species. More recently, a wide range of horizontal pictures of terrestrial environments have been made available online by different services such as Google Street View (available on http://earth.google.com). Street view imagery provides millions of geo-referenced panoramas along worldwide roads obtained using car-adapted cameras. To date, only two studies using this free source of data in biodiversity sciences are referenced in the Web of Knowledge database (‘Google Street View’ search, http://webofknowledge.com), both of which focus on animal species. In the first of them, the authors successfully identify the nesting habitats of two cliff-nesting vultures through ‘virtual’ prospection [16]. More recently, cross-validation between field and virtual occurrence data of the pine processionary moth, *Thaumetopoea pityocampa*, has demonstrated the robustness of data collected using street view imagery (SVI) [17]. Overall, these seminal works suggest that further applications of street view data should be possible from such a widely distributed source of information. Indeed, most of these pictures capture information on the surrounding vegetation, and the high quality of this imagery should allow the identification of many plant species and habitats. SVI data may thus offer a unique opportunity to massively improve distribution data on a variety of taxa at very limited cost. The condition for an efficient utilisation of such data remains, however, to be investigated.

Here, we propose a systematic approach to test whether SVI data can improve upon our knowledge of the distribution of plant species on large spatial scales. For the present discussion, we have chosen to investigate the distribution of *Arundo donax* L. (Poaceae) in continental Spain as a model system. The specific aims of this study are (i) to use a structured prospective method using aerial photographs and SVI in order to collect a large sample of species presence-absence data, (ii) to verify the reliability of distribution data collected via SVI against historic field occurrence data collected over several decades, and (iii) to investigate the impact of SVI presences and absences *vs*. field presences and (pseudo-) absences on the goodness-of-fit and outputs of SDMs. We conclude by providing, as a guideline for future applications, a critical review of the advantages, biases, and potential perspectives associated with this alternative prospection method.

## Methods

### Plant model

The giant cane, *Arundo donax* L. (Poaceae), is a perennial reed native to the Middle East. This species also occurs in other warm regions around the world, where it is usually considered an invasive species. Once considered a neophyte from Eurasia, this riparian grass has been recently designated an archaeophyte (*i*.*e*. ancient introduction) to the Mediterranean Basin, which would make it one of the oldest invasive species ever studied [10]. Due to its strong rhizomatous growth, this taxon forms dense patches of tall and robust culms (up to 6 m) in open landscapes. These sea-green culms possess about 20 alternate and nodding leaves, a plumose panicle, and secondary ramifications. Although it is possible to confuse it with some herbarium specimens (*e*.*g*. *Phragmites* sp.), the identification of *A*. *donax* is easy for the experienced eye and does not require close *in situ* observation. Its ruderal and competitive abilities allow it to invade riverbanks and agricultural margins in many warm regions worldwide [10]. This highly clonal species is mainly vegetatively dispersed through human activities and river floods. As a consequence, the species also occurs along roads and railways within its distribution area. Commonly found in warmer climates, the giant cane is known to be sensitive to low temperatures. As such, its vegetative growth is generally reduced at temperatures below 17.5°C [18]. This geophyte plant species also favours high water availability during the growing period, which explains its affinity for riparian areas.

### SVI prospection method

The proposed SVI prospection method was tested in mainland Spain, a region that fulfils the following criteria: (i) it hosts *A*. *donax* as a common species in a substantial part of the territory; (ii) it has well-maintained and reliable occurrence data from the Spanish botanical national database *Anthos* (www.anthos.es, Fundación Biodiversidad, Spain). A total of 505 geo-located records of *A*. *donax* occurrences dating back as far as 1839 were extracted from the *Anthos* database. The sampling strategy for the SVI prospection was based on a simple random design. The study area was divided into a grid of 5484 6-arc-min (*i*.*e*. 0.1x0.1 degree) cells using Quantum GIS 2.4 (QGIS, www.qgis.org). Cells with >25% of their surface area in the sea or otherwise outside of Spain’s borders were excluded from the analysis. We randomly selected 20% of the cells (N = 1097) using the QGIS-function 'Random selection' for sampling by the SVI prospection method. Of the 505 field occurrences from the *Anthos* database we reduced to only 216 cells with field presence. By chance, ten of these happened to also be among the original set of 1097 previously selected cells. The remaining 206 cells with *Anthos* records were also prospected in order to evaluate the omission rate of SVI data.

A repetitive procedure was conducted in order to standardize the SVI prospection for each cell. The procedure involves the following three steps (S1 Video).

**Fig 1:**
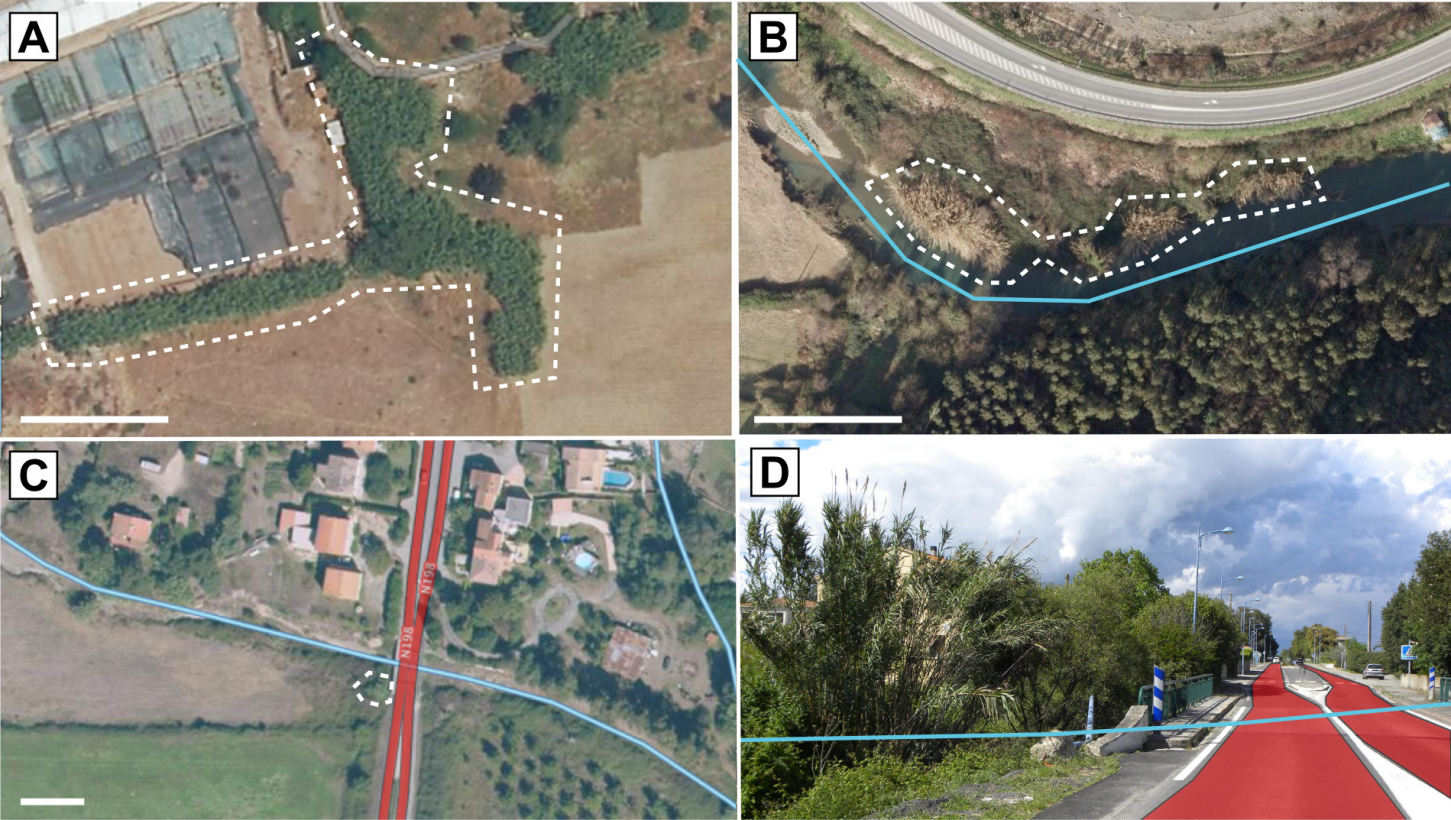
Species identification with SVI prospection. Detection of suitable habitats for *Arundo donax*, *e*.*g*. farmland margins (A) and riversides (B), or graphic patterns on aerial photographs. When viewed by vertical perception, *A*. *donax* forms radial to linear black-spotted patches with a sea-green colour after the growth season, and a light brown colour following winter. (C) Vertical localization of a suitable habitat (riversides, blue line) next to a street view road linear (red lines; 41.9639°N, 9.3973°E), and (D) horizontal projection on street view imagery allowing identification of *A*. *donax* (on the left). Scale bar = 20 m.

(i) Explore each cell eastward and southward, using aerial photographs scaled between 1:1x10^5^ and 1:1x10^4^ (*c*. 1–10 km a.s.l.), to look for suitable areas for *A*. *donax* (*e*.*g*. human-disturbed rivers, farmlands and suburban zones) that include street view transects (*e*.*g*. river bridges).
(ii) Detect potential occurrences between the 1:1x10^4^ and 1:1x10^3^ scales (*c*. 0.1–1 km a.s.l.) using graphical insights based on plant aspect (*e*.*g*. sea-green linear or radial black-spotted patches along stream or field edges—Fig 1A, 1B and 1C).
(iii) Control for species presence using street view horizontal perception (Fig 1D). Cell prospection was halted when one individual of *A*. *donax* was unequivocally identified, or after a maximum of 5 min of prospection had elapsed with no positive identifications. This was done to keep the overall prospection duration within reasonable limits, which generally worked well enough for our preliminary tests but must be adapted in further studies on other species. Indeed, the first prospective step on aerial photographs could be more time-consuming, *e*.*g*. for small non-spread taxa distributed in less easily identifiable habitats than riparian or ruderal zones. Consequently, an absence was reported as having occurred in the entire prospected cell (and thus located at its centroid), whereas for each occurrence an accurate geographical coordinate is given. For this study, the SVI prospection lasted 50 hours, which was spread over one working month with 2–4 hours of prospection per day in order to avoid diligence failure and other bias linked to such repetitive work.

### Assessment of SVI data and method reliability

To evaluate the omission risks of SVI prospection, we started by evaluating the difference between data collected on SVI and data collected in field as a percentage of mismatch in the cells that showed field occurrence. Next, we tested the hypothesis that mismatches between SVI and field data are linked to the density of roads covered by SVI within each prospected cell: fewer roads may be found within non-congruent cells than within congruent ones. The Google SVI network is currently not available in GIS vector format. Consequently, we postulated that the exhaustive road network is closely correlated to the SVI network among continental Spain. We exported the continental Spain road network from OpenStreetMap Data (http://download.geofabrik.de/) to estimate the total length of road (in km) per cell. A Generalized Linear Model (GLM) with a binomial distribution of error and a logit link was performed in order to test the influence of road length per cell on the probability of match between field and SVI data. This response variable was defined as 1 for a match between both methods and 0 for each SVI-determined absence that corresponded to an occurrence in the *Anthos* database. Road length was log-transformed.

### Comparison of SVI and field data in SDM

Distribution (probability of presence) of *A*. *donax* was assessed using a Generalised Linear Model (GLM), with a binomial distribution of error and a logit link [19]. This method gathers several explanatory variables in a linear function in order to model a binary response ranging between 0 and 1. We compared the outcomes of SDMs built with either field data or SVI data. Three different datasets were used: (i) *Anthos*: 216 field presences from the *Anthos* database and 216 pseudo-absences data randomly selected among the 5268 remaining cells (with 1000 replicates), (ii) *SVI*_sub_: a subset of 216 presences and 216 absences randomly selected from the entire SVI dataset (with 1000 replicates), and (iii) *SVI*_full_: the complete SVI dataset. The first dataset can be thought of as the means to a fundamental assessment of SVI data, as it represents the basic model of species distribution that can be performed using data from a botanical database. Because of its similar sample size, the second dataset allows a comparison of model efficiency between field data and SVI data. The third dataset illustrates the type of species distribution modelling that can be performed using SVI data, taking into account the effects of both data quality (as model 2) and quantity.

We used a set of three synthetic climatic data as explanatory variables in the GLM. Climatic data were extracted from the Bioclim dataset, provided by WorldClim v.1.4 [20], which includes nineteen climate variables in a GIS-based raster format. A principal components analysis (PCA) using values from the nineteen climate variables for each cell was performed to produce an uncorrelated set of three synthetic variables, hereafter referred to as PC1, PC2, and PC3 (the values of the three first principal components). PC1 was found to be negatively correlated with the temperature-related bioclimatic variables (Max Temperature of Warmest Month (BIO5) and Mean Temperature of Warmest Quarter (BIO10)), and positively correlated with precipitation-related variables (Precipitation of Driest Month (BIO14), Precipitation of Driest Quarter (BIO17) and Precipitation of Warmest Quarter (BIO18)). High values of PC1 therefore indicate a temperate and wet climate whereas low values of PC1 indicate a warm and dry climate. PC2 was negatively correlated with the bioclimatic variables linked to cold stress (Min Temperature of Coldest Month (BIO6), Mean Temperature of Coldest Quarter (BIO11) and Precipitation of Coldest Quarter (BIO19)). High values of PC2 indicate a high level of cold stress during winters with low precipitation. Finally, PC3 was negatively correlated with Mean Temperature of Wettest Quarter (BIO8) and positively with Isothermality (BIO3). High values of PC3 indicate a cold climate during wet periods and a higher degree of temperature fluctuation within each month than over the course of the year (S1 Fig). The PCA was generated using the *ade4* R-package in R 3.2.2 [21].

## Results

**Fig 2:**
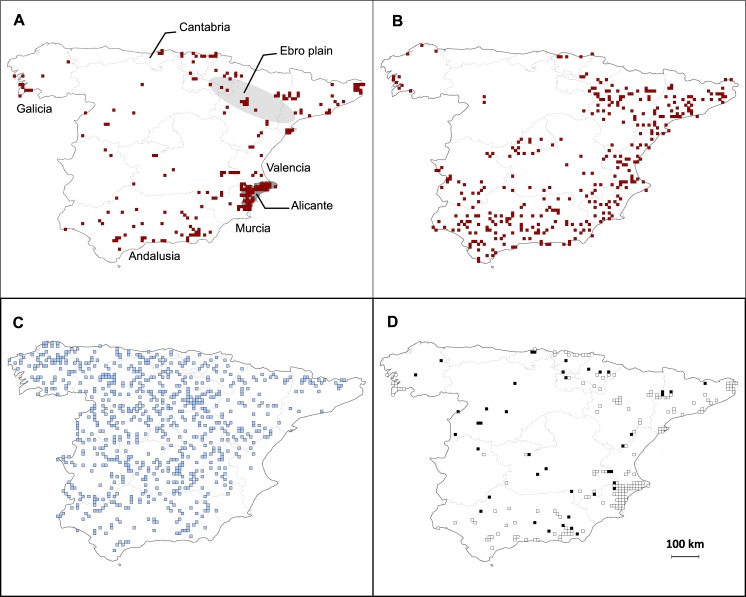
Geographical distribution of *Arundo donax* in Spain. Distributions of (A) field presences from classical field-based database *Anthos* (216 cells), (B) presences (345 cells) collected using SVI method (including only randomly selected cells), (C) absences (752 cells) collected using SVI method and (D) matches (white cells) and mismatches (black cells) between field (*Anthos*) and SVI presences.

The 216 occurrences of *A*. *donax* derived from the field data were mainly located along the Mediterranean coast, from the coastline to about 100 km inland, with a deeper continental occurrence along the Ebro alluvial plain (NE Spain; Fig 2A). Occurrences were also recorded in two Atlantic regions (Cantabria and Galicia), as well as other regions scattered sporadically over the greater inland area. An important part of the data was obviously located close to towns, especially between Valencia and Murcia. The SVI prospection collected 345 occurrences of *A*. *donax* among the 1097 sampled cells (31%; Fig 2B). The species was not recorded, and therefore considered absent, from the 752 remaining prospected cells (Fig 2C). On a coarse scale, species potential distributions obtained from these two sources of data were roughly similar. On a finer scale, SVI provided relatively more data further inland, as well as a much denser potential distribution alongside the Ebro valley and in Andalusia.

**Fig 3:**
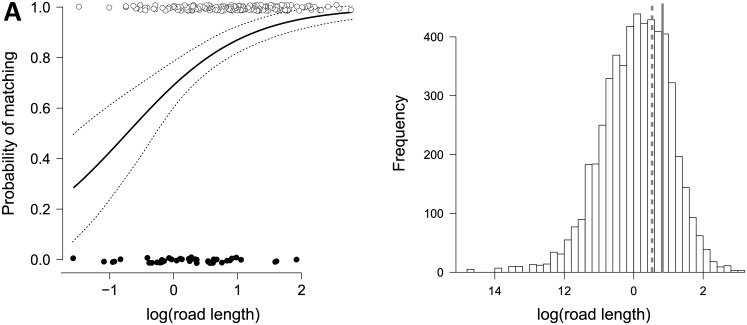
Road length effect on mismatch between field and SVI data. (A) The effect of road length on the probability of matching occurrence of *Arundo donax* in Spain between field-collected data (*Anthos*) and data collected from street view imagery (white dots, data matches; black dots, data mismatches). The dotted lines delimit the 95% CI area; (B) frequency distribution of log-transformed road length across Spain (N = 5484) with the continuous line indicating the median value of road length within the field dataset. The dashed line indicates the road length value for which a predicted probability of matching reached 80%.

The SVI prospection detected *A*. *donax* in 81.1% of the 216 cells that included field presences (Fig 2D). The mismatches between SVI and field occurrences are mainly localized to inland cells, *i*.*e*. in regions where few occurrences have been recorded by both methods. The probability of matching between the two sources of data was positively influenced by the total length of roads (on a log scale) available in the cell (*β* = 1.10 ± 0.25; Fig 3). However, the proportion of deviance explained by this model remains small (11%), suggesting that other factors might be influencing the mismatches. Furthermore, median road length (on log scale) across all 5484 cells was 0.13, a value for which the predicted matching probability was as high as 72%. Interestingly, the median road length (on log scale) for the cells with field data (*Anthos* database) was much greater (0.83) than the one across all of Spain (median for SVI data: 0.08; Fig 3).

**Fig 4:**
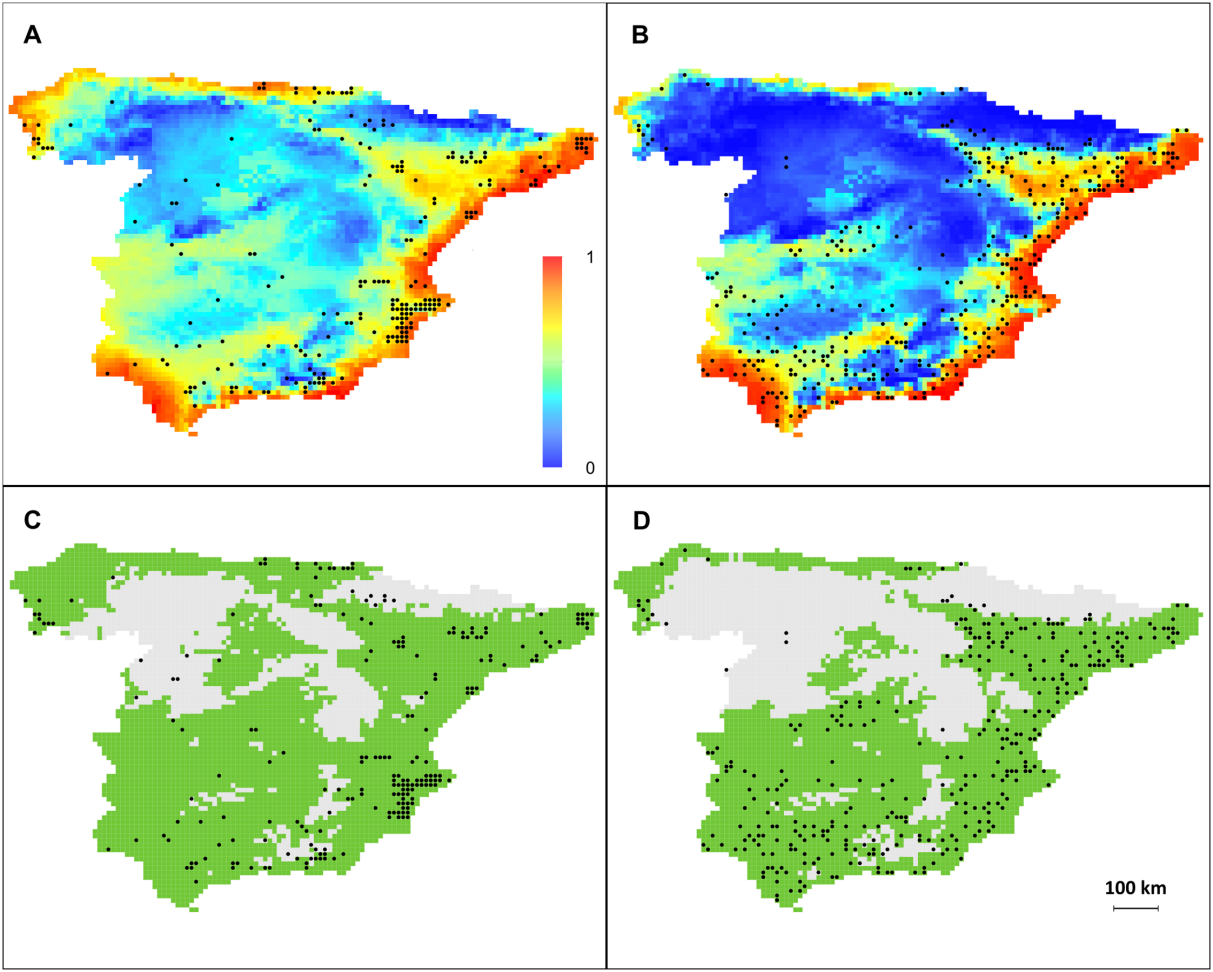
Modeling of *A*. *donax* distribution using SVI and field data. Presence probability of *Arundo donax* (upper panel) and binary prediction (lower panel) of presence (red) and absence (blue) across Spain, as predicted by the GLM using (A, C) field data (*Anthos*, N = 216 presences and 216 pseudo-absences) and (B, D) SVI-collected data (*SVI*_*full*_, N = 345 presences and 752 absences). *SVI*_*sub*_ outputs were highly similar to *SVI*_*full*_. Continuous probabilities of presence (A, B) were converted into binary prediction (C, D) following the 10% threshold method, *i*.*e*. the minimum probability for presence cells after discarding the 10% lowest values (0.31 for field-data fitted model and 0.14 for SVI-data model).

**Fig 5:**
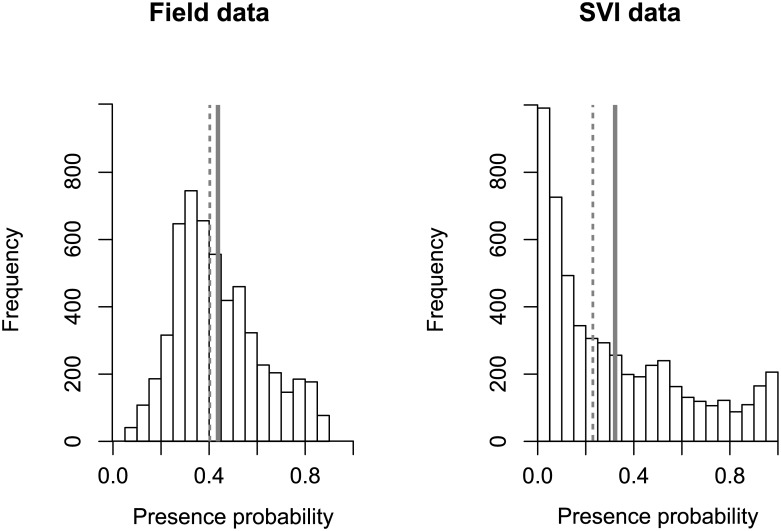
Presence probabilities of *A*. *donax* using SVI and field data. Frequency distributions of presence probability values across the 5484 cells covering Spain, as predicted by the GLM based on field data (left panel; *Anthos* N = 216 for presences and 216 pseudo-absences) and SVI data (right panel; *SVI*_*full*_, N = 345 presences and 752 absences). Dashed and continuous lines indicate median and mean presence probabilities, respectively.

The proportion of deviance explained by models using SVI data was three times greater than that of models with field presence data and randomly selected pseudo-absences (ca. 33% *vs*. 11%; table 1). The three models retained the three synthetic bioclimatic variables as significant (effect size > 0). However, effect sizes (in absolute values) associated with bioclimatic variables were two to five times greater in SVI-derived models than in field-based ones. This discrepancy did not arise from a difference in the number of absence data used in the model. Indeed, randomly selecting 216 presence and 216 absence data, so as to match the sample size of field-based data, provided results very similar to those obtained by considering the entire SVI dataset (Table 1). For all of the predictive variables that were considered, we found no overlap between the confidence intervals of the predicted slopes from SVI (*SVI*_full_ or *SVI*_sub_) and from field-data (Table 1), further suggesting that the model based on field data had greatly underestimated the effect of bioclimatic variables. Projection of models based on field data and SVI data produced a similar potential distribution across Spain (Fig 4), with highly suitable areas along the coasts, and moderately suitable areas along the Ebro valley and north and south of the Sierra Morena Mountains, up to Madrid. However, the potential distribution model based on SVI data was more conservative. For example, the model based on field data predicted the arid and cold central Spain (in Castilla y Leon) and the Sierra Morena mountains to be poorly suitable, whereas the full SVI model predicted those areas to be unsuitable. The latter result appears more consistent with the known ecological requirements of the species. This was confirmed by the frequency distributions of probabilities of presence across Spain predicted by both models (Fig 5): no cell was predicted to be unsuitable with the model based on field data, which found a median probability of presence across Spain of 0.4. By contrast, the model based on SVI data predicted a higher proportion of unsuitable cells and a lower median probability of presence (0.22).

**Table 1:** Proportion of deviance explained and effect size (95% confidence intervals into brackets) for three predictive variables (3 first axes of a PCA run on bioclimatic variables, see Methods) by models exploring the distribution of *Arundo donax*. The 216 absence data used in (i) and (ii) were randomly generated 1000 times and the results shown refer to median values.

Data used to fit GLM	% Deviance explained (±SD)	PC1 effect size	PC2 effect size	PC3 effect size
(i) 216 field presences & 216 pseudo-absences	11.3 ± 2	-0.08 [-0.007; -0.15]	-0.22 [-0.12; -0.31]	-0.43 [-0.29; -0.57]
(ii) 216 SVI presences & 216 SVI absences	33.6 ± 3	-0.39 [-0.50; -0.28]	-0.44 [-0.60; -0.31]	-1.02 [-1.25; -0.80]
(iii) 470 SVI presences & 760 SVI absences	32.7	-0.39 [-0.46; -0.32]	-0.41 [-0.49; -0.33]	-1.02 [-1.16; -0.87]

From an ecological perspective, the probability of presence of *A*. *donax* was predicted to be higher where temperatures are mild during wet periods and thermal fluctuations are moderate (i.e. for lower values of PC3). The probability of presence was also elevated where limited cold stress was coupled with high water availability in winter (i.e. for lower values of PC2), and where warm and dry climates were found (i.e. for lower values of PC1).

## Discussion

The more striking outcomes of our alternative prospection method using street view imagery (SVI) were the impressive amount of presence data collected in a relatively short time, and the fact that it provided absence data (which are not available in field databases). This has made it possible for us to model different ecological niches (with drastic changes in effect size for the three considered predictive variables) and spatial distributions for the species in question.

We collected, using SVI, 345 presences, *i*.*e*. more than 1.5 times as many as the 216 field presence records in the *Anthos* national Spanish database. In addition, our SVI data were collected by a lone worker in about 50 hours (parsimoniously spread over one working month) without incurring any travel expenses. By contrast, the traditional field dataset has been amassing since 1839 and has involved the work of hundreds of botanists. Our random selection of prospected cells limited data aggregation within over-informed regions, *e*.*g*. around major cities, research centers and university campus, or within well-prospected regions. For example, 22% of field presences are located in the Province of Alicante, where SVI prospection localized only 8% of SVI presences (Fig 2A); this prospection bias is specific to the *Anthos* database, and mainly due to the detailed prospection of the Province of Alicante through the PhD thesis of Luis Serra Laliga, generating a regional database of more than 100 000 species records [22]. Moreover, the SVI data are both more homogeneously distributed and more accurately located than field data: each presence is tied to an accurate GPS position (*vs*. several field data corresponding to centroids of regional locality). Consequently, anyone can verify the SVI presence using our sampling information (S1 Table). These data can then be used for diachronical studies using SVI methods of field monitoring.

The exploration of the 216 cells gathering *Anthos* field presences with the SVI prospection method confirmed species occurrence in 81.1% of cells. This implies a mismatch proportion of 18.9%, which though weak is not null, suggesting a significant impact due to false absences in SDMs [23]. Indeed, false absences could limit the informative power of environmental models of a given species’ ecological niche. However, in our study case, it appears that mismatched cells do not correspond to occurrences located at the environmental range periphery [24]. Consequently, the environmental ranges captured by both sources of species occurrences appear quite similar (S2 Fig). There are three main hypotheses that could be advanced to explain the mismatches, and they are as follows.

1. Considering the dates of observation in the *Anthos* database, which span the years 1839 to 2014, the extinctions of formerly observed populations could explain some mismatches. It is noteworthy that 44% of the mismatching cells correspond to occurrences recorded before the 1990s in the *Anthos* database (28% for the matching cells) and that no mismatch was noticed for field data recorded after 2010 (two records for the matching cells).
2. Mismatches could be due to a lack of SVI in cell, leading to an indication of apparent species absence in a poorly prospected cell. This hypothesis is supported by the fact that 59% of the mismatching cells (N = 24) exhibit a total road length (on a log scale) that is below the 0.45 threshold that corresponds to an 80% probability of matching.
3. Finally, these mismatches could be explained by their eco-geographical position in the edge of the bioclimatic niche of *A*. *donax*. Indeed, field presences could over-inform species occurrence in its limits of distribution and ecological niche: one can expect that a naturalist is more likely to mention a species when this species is rare in this area. This is actually in line with the fact that most mismatches were inland, where both SVI and field presences are rare (Fig 2D).

If field prospection overestimates rare occurrences, SVI plant prospection may underestimate them. Consequently, this alternative method must be led on common species to minimize false absences. A current limit of the SVI prospection is of course its restriction to the vicinity of the road network. Interestingly however, we showed that field data were more highly biased than SVI data toward cells with a denser road network, a pattern likely to be common to most field databases and that undermine their value in the SDM context too.

A crucial added value of the SVI method is that it provides absence data that are supported by a standardised prospection method and a random sampling strategy (at the level of the study area). Biogeographical studies often misinterpret the absence of occurrence data in a region as a species absence, an effect often referred to as 'pseudo-absence' [25]. This inference supposes that field presences exhaustively describe species distribution, a postulate that is rarely met. The impact of pseudo-absence on a model's discriminating ability can be mitigated by using alternative pseudo-absence selection strategies (*e*.*g*. by using a minimal and maximal distance to presence points for selecting pseudo-absence candidates, or by defining environmental conditions in which pseudo-absences can be selected which differ from the environmental conditions of a defined proportion of presence data). However, these methods may lead to over-optimistic model evaluations and they remain in all cases arbitrarily selected. Spatial homogeneity in coverage (achieved by a randomized sampling effort) is indeed a key to modelling species distribution [26]. Classic methods involving the arbitrary selection of pseudo-absence can therefore define pseudo-absence in areas where the species was not actually looked for. By contrast, the SVI method defines absence data by a prospection similar to the one used for recording presence data. In our case, the results clearly show that absence data collected using the SVI method changed the model outputs relative to presence-pseudo absence data, thus improving discrimination between presence and absence, and decreasing the probability of occurrence of the giant cane in cold and arid areas. We observed that even for a similar sample size, SVI-based GLMs showed an explained deviance three times greater than field-based models, with substantially higher effect sizes associated with bioclimatic variables. This large difference between the two types of data may putatively be attributed to false absences included in pseudo-absence data related to the SVI data. False negatives are indeed particularly deleterious to model calibration and data fitting [23]. It is worthwhile to note that the proportion of deviance explained by SVI-based models using the whole dataset or a subset of it (with less than half of the total sample) were highly similar. This result suggests that, in the case of *A*. *donax*, the SVI prospection in 10% of cells covering Spain would provide a similar goodness-of-fit for model calibration while increasing the time and cost effectiveness of the SVI proposed method.

The SVI method has the potential to improve the accuracy of characterizations of species distribution and therefore can be highly valuable for *e*.*g*. defining protected areas or areas subject to the spread of an invasive species. As a result of their affinity for human-disturbed areas, alien species are particularly well-suited models for detection via street view imagery. Indeed, roadsides are often privileged vectors of dispersion for invasive plant taxa [27]. Besides, most of these species possess specific morphology (*i*.*e*. shape, leaf form, inflorescence), which makes their identification easy. For example, we noticed during SVI prospection interesting variations in the occurrence of taxa such as tree of heaven (*Ailanthus altissima* (Mill.) Swingle), pigfaces (*Carpobrotus* N.E. Br) and Barbary fig (*Opuntia ficus-indica* (L.) Mill.). SVI could also be used to detect and track the zoning and dynamics of plant communities, to map specific habitats, to detect and map specific plant hosts, or to better inform the land cover acquisition process or the habitat state of conservation. In addition, when *in situ* sampling is required, street view imagery can be very useful in maximising the success of sampling campaigns. For example, we firstly used this SVI method to pre-localize one third of the localities of the overlooked *Arundo plinii* s.l. before collecting it in the Mediterranean [28]. Besides occurrence data, the SVI method can also be modified to estimate species relative abundance by subsampling road transects.

However, the deployment of the SVI prospection method must be undertaken with care by taking into account the following intrinsic limitations: (i) the sampled object (species, habitats or damages marks) must be highly recognisable in all seasons, (ii) the prospection is restricted to roadsides, which implies that (iii) it may under-detect occurrences where the species is rare. For example, when it comes to plant species, we recommend that the SVI-prospection method be applied only to highly recognizable common taxa such as trees, shrubs or tussock herbs, which maintain a characteristic shape throughout the year. The latter consideration is crucial: special attention must be paid to seasonal differences in species’ morphological aspects (*e*.*g*. colour phenological differences of *A*. *donax* leaves; Fig 1A and 1B) or to the periodicity of physical damage.

Eventually, the ecological restriction of street view to roadsides will be partly removed when its coverage is extended into the wild. Indeed, this has already begun with street view imagery now beginning to depict natural areas such as national parks (*e*.*g*. Grand Canyon, USA; Galápagos Islands, Ecuador). This new data collection will allow naturalists to virtually visit many natural landscapes, making SVI prospection a boon to future biodiversity studies.

## Supporting Information

S1 Fig Geographic distribution of Bioclim PCA. Distribution of the three synthetic bioclimatic variables PC1 (A), PC2 (B) and PC3 (C) used to fit the GLM across Spain.
(EPS)

S2 Fig Environmental range for SVI and field data. Environmental range captured by field-collected (*Anthos*, upper panel) and SVI-collected (Street View, lower panel) occurrences: density distribution of environmental variables PC1 (left panel), PC2 (central panel) and PC3 (right panel) across presence locations.
(TIF)

S1 Table Dataset information. Geographical coordinates of prospected cells, with presence data from the *Anthos* database (*Anthos*), presence or absence data from SVI prospection (SVI), and accurate geographical coordinates of SVI presence (PresenceX, PresenceY). The ‘type’ column indicates the use of data in GLMs and in validation against field data.
(TXT)

S1 Video Tutorial of SVI prospection in a cell. (MP4)
