# Supplementary figures and images for: Species Distribution 2.0: An Accurate Time- and Cost-Effective Method of Prospection Using Street View Imagery
Source: PLoS One. 2016 Jan 11;11(1):e0146899. doi: 10.1371/journal.pone.0146899 (PMC4709242; doi:10.1371/journal.pone.0146899)

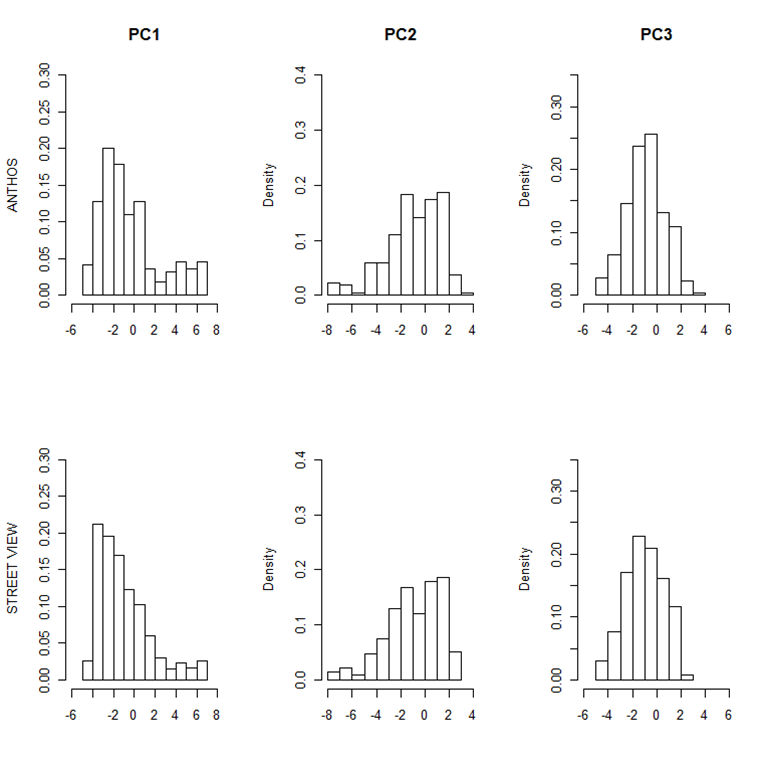

Supplement: S2 Fig — Environmental range captured by field-collected (Anthos, upper panel) and SVI-collected (Street View, lower panel) occurrences: density distribution of environmental variables PC1 (left panel), PC2 (central panel) and PC3 (right panel) across presence locations. (TIF) [file pone.0146899.s002.tif]
